# Prevalence and Triggering Factors of Headache among Jordanian Adolescents in Al-Mafraq Region

**DOI:** 10.1155/2023/5548694

**Published:** 2023-11-13

**Authors:** Mohammed Albashtawy, Nisser Alhroub, Zohair Zafar, Shaher Hamaideh, Laith Al-Osoufe, Malakeh Malak, Mahmoud Bashtawi, Asem Abdalrahim, Ahmad Rayan, Sa'd Albashtawy, Abdullah Alkhawaldeh, Ma'en Aljezawi, Mohammed Jallad, Imad Abu Khader, Bayan Albashtawy, Muna Al-Kharabsheh

**Affiliations:** ^1^Department of Community and Mental Health, Princess Salma Faculty of Nursing, Al al-Bayt University, Mafraq, Jordan; ^2^Faculty of Nursing, Jerash University, Jerash, Jordan; ^3^Department of Emergency Medicine, King Fahad Hospital Jeddah, Jeddah, Saudi Arabia; ^4^Community and Mental Health Nursing Department, Faculty of Nursing, Hashemite University, Zarqa, Jordan; ^5^Department of Pediatric Nursing, Faculty of Nursing, Jerash University, Jerash, Jordan; ^6^Department of Community Health Nursing, Faculty of Nursing, Al-Zaytoonah University of Jordan, Amman, Jordan; ^7^Department of Neuroscience, Faculty of Medicine, Jordan University of Science and Technology, Irbid, Jordan; ^8^Faculty of Nursing, Zarqa University, Zarqa, Jordan; ^9^Ministry of Health, Amman, Jordan; ^10^College of Nursing, Sultan Qaboos University, Muscat, Oman; ^11^Adult Health Nursing, Faculty of Graduate Studies, Arab American University, Jenin, State of Palestine; ^12^Nursing College, Al-Balqa Applied University, Salt, Jordan

## Abstract

**Aims:**

This study evaluates the epidemiology of headache and migraine among adolescents aged 12 to 15.

**Methods:**

A school-based cross-sectional study was conducted to collect and analyze data from students in grades 7–10 over the course of one month, using a simple random sampling method. The overall number of participants in this study was 692, with an average age of 13.9 years (SD = 1.3). Descriptive measures and Fisher's exact test were computed. Multivariate regression was calculated to assess the predictors of headache and migraine. *Findings*. Approximately one-half of the students reported having headaches: tension-type headaches (10.3%), migraines (4.8%), and other headache types (31.5%). Moreover, girl students in the age group of 14-15 reported more headaches and migraines.

**Conclusion:**

The prevalence of headache and migraine in Jordan is high and increasing as students grow older. Health education programs led by school nurses and other healthcare practitioners are urgently needed.

## 1. Introduction

Headache is one of the common health problems that affect focus, performance, and overall quality of life. It is a common complaint that affects people of all ages [1, 2]. Headaches not only affect individuals' quality of life and activity levels, but they also increase society's economic burden over time [3, 4]. Furthermore, in adolescents, they can lead to increased stress, absenteeism, a reduction in day-to-day activities, and academic achievement, as well as difficulty in forming and maintaining social relationships [3, 5–7]. Migraine has been designated by the World Health Organization (WHO) as one of the world's most incapacitating conditions [8, 9].

Primary headache is a major health concern in many countries, and it is one of the most common disorders worldwide [10, 11]. In developed countries, it is estimated that nearly a tenth of the population is affected [11], and the same results have been reported in developing countries, including Saudi Arabia [12, 13], Oman [14, 15], Jordan [16], Qatar [17], and Yemen [18]. Primary headache is also a major health concern and one of the most common diseases among adolescents around the world, leading to the use of over-the-counter medications, overdosing, and self-medication, all of which can lead to substance abuse [3, 19].

A tension-type headache is one of the most common types. It can be acute or chronic, and it has significant social implications. On the other hand, in rare cases, when chronic progressive headaches occur along with other neurological symptoms, recurring headaches can be a sign of a more serious medical condition, such as hemorrhage, encephalitis, meningitis, or tumor [20].

Migraine is characterized by a pulsating headache on one side of the head, as well as phonophobia and photophobia. Migraine conditions rank eighth in terms of their impact on people's quality of life and on society [21, 22]. According to the International Headache Society (IHS) and other international studies [20], it appears to be influenced by a variety of factors, including age, physical and mental stress, eating habits, and genetic factors [3, 10, 23]. Moreover, according to Vos et al. [24], migraine is the leading cause of headaches worldwide and is identified among the top causes of disability. Numerous studies have shown that migraines affect people of all ages, income levels, races, social status, and local regions. Furthermore, psychological traits (type of person) are thought to play an important role in headache and migraine by acting as irritants [3, 22, 25]. Migraine sufferers usually experience an aura for half an hour before the actual pain begins. It has temporary neurological symptoms that include visual disturbance followed by headache. The type without an aura has common symptoms, such as a sudden onset of joint pain [26, 27].

In Jordan, a few studies have linked the problem of headaches and migraines over the past two decades, especially among children and adolescents (Alawneh and, Bataineh, 2006; [28, 29]. For example, Alawneh and Bataineh (2006) studied the problem among school-aged children aged 6–14 in 2005 and found that one out of every four children had headaches, and about 3% had migraines (Alawneh and, Bataineh, 2006).

Another study conducted by ALBashtawy et al. found that approximately two-thirds of high school students reported experiencing headache. 19.0% reported tension, 8.8% migraines, and 39.0% an unknown type [28]. However, few studies on headache and migraine have been conducted in Jordan over the years, and this will be the first among adolescents, according to the best information available. Researching headache can improve our understanding of the condition and lead to more treatments that are effective. Education campaigns can raise public awareness of headaches, their causes, and the importance of receiving treatment as soon as possible. Evaluating the frequency and contributing factors of headaches is crucial to an adolescent's general quality of life, short- and long-term health, and well-being. It makes an early detection, intervention, and support easier for this age group, which enhances the quality of life and health outcomes. As a result, this study was carried out in Jordan to assess the prevalence and triggering factors of headache among adolescents aged 12 to 15.

## 2. Research Questions

The current study was conducted to answer these questions among adolescents aged 12–15:

1. What are the characteristics of headache among adolescents in school?
2. What is the prevalence of probable headache, tension-type headache, and migraine among adolescents in school?
3. What medical help is available for headache among adolescents?
4. What are the triggering factors for headache among adolescents in school?

## 3. Methods

### 3.1. Study Design and Population

A school-based cross-sectional study was conducted to collect and analyze data from students in grades 7–10 (12–15 years old) over the course of one month.

Using a simple random sampling method, the participants were chosen from eight schools in Mafraq Governorate. In the designated schools, all adolescent students in grades 7–10 (754) were asked to participate.

The exclusion criteria for the study were students who do not fall within the 12–15 age range, students who decline to participate in this study, students who have not obtained signed parental consent, and any acute or chronic condition that would limit the ability of the student to participate in the study.

Sampling: *G*^*∗*^ power software was used to identify the minimal sample size for this study, with a confidence interval of 1.96, a margin of error of 5%, and the probable prevalence of headache 50%. The minimal effect sample size calculated was 387 [30]. 754 students were initially invited to participate in the study in order to compensate for those not having signed parental consent or incomplete or missing data; in addition, many students were interested in taking part.

A week before collection of the data, children were given letters that clearly explained the survey objectives, the questionnaire, and two consent forms to take home. Students and parents were each asked to sign a form of informed consent and an informed permission form. Students who submitted completed consent and assent sheets on the day of data collection were invited to participate.

The survey was carried out in the students' classes at their respective schools. The adolescents were advised that participation was entirely voluntary and that they might opt out at any time. Six hundred and ninety-two of the 754 students who were initially invited to participate in the study agreed, giving the study a 91.8% response rate. The participants were asked to fill out a questionnaire on their headache and migraine symptoms during the previous month, January to February 2020.

The questionnaire was modified and adapted from previous studies [16, 18, 28, 31, 32]. It was then revised by six academics with expertise in neurology, pain management, and public health, before being sent to the other research members for final feedback. The final draft of the questionnaire was tested in a pilot study with 39 students to determine its face and content validity. The pilot was intended to identify any anticipated problems or obstacles to the data collection procedures. In addition, it helped determine possible response rates as well as the time required to complete the questionnaire, which ranged from 15 to 20 minutes. The final comments revealed that all items were clear, comprehensive, suitable, and easy to complete.

### 3.2. Questionnaire

The first section of the questionnaire collected sociodemographic information, such as age, gender, family monthly income, and mothers' and fathers' education levels. The second section (11 questions) covered the following topics: the type of headache, intensity and duration of pain, time of headache, associated characteristics, absence from school, daily activity, family history of headache, seeking medical help for headache, treatment modality, and analgesic advice. The third section covered the common migraine symptoms, and the fourth section covered the triggering factors for headaches. The questionnaire was followed by clinical interviews according to IHS standards (3rd edition, beta version) which are widely used and approved in epidemiological surveys around the world and was based on the expertise and experience of headache experts over many years [20]. It was translated from English into Arabic by a group of experts.

### 3.3. Statistical Analysis

SPSS-26 was used to enter and analyze the collected data. The Shapiro test was used to test the normality of the data. The authors calculated the frequencies, means, and standard deviations. Fisher's exact test was used to show the differences between the variables, with a *p* value of 0.05 considered significant. Moreover, multivariate regression was calculated to assess the predictors of headache and migraine.

### 3.4. Ethical Considerations

Permission was first obtained from the government university's Ethical Approval Committee and then from the Mafraq District Education Office. Furthermore, verbal permission from the administration of the nominated schools was gained.

## 4. Results

### 4.1. Characteristics of Study Participants

The findings demonstrated that the data had a normal distribution. There were 692 participants in all, 358 (51.7%) boys and 334 (48.3%) girls among them. They were between 12 and 15 years old, with an average age of 13.9 years (SD = 1.3). Half of the participants (53.8%) had a family income of more than $500 per month. Over half of the mothers (51.9%) and over two-thirds of fathers (70%) had completed secondary or higher education (see Table 1).

### 4.2. Prevalence of Headache Types among Adolescents

Table 2 shows that approximately one-half of the students reported having headaches, tension-type headaches (10.3%), migraines (4.8%), and other headache types (31.5%). Moreover, female students and the age group 14–15 reported more headaches and migraines than male students and the lower age group (12–13), with a statistically significant difference.

### 4.3. Characteristics of Headache among Adolescents

In terms of headache characteristics, over two-thirds (69.9%) of respondents experienced a headache in the afternoon, with 17.1% experiencing severe discomfort. Nearly half (48.1%) reported persistent pain lasting 2 to 72 hours, with one-fifth describing it as gradually developing (20.2%), intense pain (16.5%), and more than 15% as difficult to explain. Furthermore, they reported that pain severity limits their daily activities (32.6%) and prevents them from attending school (60.2%). Approximately 45.4% reported having a family history of headache, with 13.4% reporting that their fathers had a history of headache. About one-fifth of the students sought help for headaches, and medication was recommended for 20%, with nearly two-thirds reporting using sleep as a first-line treatment for headaches and migraines.

Analgesics were recommended for 50.0% of students by their families and 14.4% by pharmacists. The major symptoms associated with pain were intensity increased with physical activity (68.0%), pulsating pain (58.0%), unilateralism (54.0%), photophobia (42.0%), and phonophobia (22.0%) (see Table 3).

### 4.4. Triggering Factors for Headaches among Adolescents

Table 4 shows that the triggering factors for headaches were increased with anxiety and stress (85.7%), too much or not enough sleep (80.7%), followed by physical exertion and fatigue (75.8%). Regarding boys, 81.5% reported that physical exertion and fatigue, followed by anxiety and stress (80.1%), were the leading triggers. On the other hand, girls reported that anxiety and stress (92.5%) and loud noises (84.2%) were among the most common headache triggers.

### 4.5. Predictors of Headache and Migraine among Adolescents

As shown in Table 5, girl students and the age group of 14-15 were the predictors of headaches and migraines.

## 5. Discussion

Headache is not just a barrier to daily life for adults. In children and adolescents, common headaches also lead to a reduction in the quality of life and limitations in social, educational, and professional fields [33, 34]. The current study assessed the epidemiology of headache and migraine among adolescents aged 12–15 during the previous month. As far as we know, this is the first study in Jordan among adolescents to shed light on this problem, which affects the learning process of children. The findings show that 46.6% of participants had headaches during the past month. This is less than previously reported figures. ALBashtawy et al. reported that about two-thirds of high school students had headaches [28]. In addition, Alzoubi et al. estimated the prevalence of migraine among adults in Jordan at 82.3% [16] and in Yemen at more than three-quarters (76.6%) [18]. In contrast, our findings were slightly higher than those of Deleu and Hanssens in Oman, where the prevalence of headache was 45% [15] and more than the global average of 45% [21]. These differences can be explained by different functional definitions and measures of headache and migraine, participants with different social characteristics, different study designs, and higher estimates of moderate pain among individuals [12].

The findings of this study should shed light on one of Jordan's biggest public health problems. Tension-induced migraine headaches affected 10.3% of students, lower than the reported rate for the adult population in Jordan (36.1%) [16] and Yemen (27.1%) [18]. In addition, our findings showed that approximately 4.8% of students complained of migraines, lower than the percentage reported in other countries (6–12%) [12, 16, 21], and below that reported in European countries (11.3%) [35].

An afternoon headache with a moderate strength of 4–72 hours was considered a major headache among high school students. According to Abdo et al., a family history of headache was reported in almost half of the participants (45.4%). One-third reported that pain affected their daily activities, and 60.2% reported that it increased their absenteeism [18]. The effects of headache on students' work and education are difficult to clarify because there may be confusing factors [36]. In research conducted in the United Arab Emirates, it was discovered that about one-third of adolescents were reported as missing school due to headache [17].

The family is considered a major source of information about analgesics and medication; approximately a quarter of students seek medical attention. Many participants in the current study used sleep as a first-line treatment, findings consistent with past studies [16, 18, 29]. This effect can be explained by the very low cost and availability of over-the-counter medication, with few side effects [16, 18].

According to other studies Alawneh and, Bataineh, 2006; [16, 18] more boys than girls reported headache. However, in the current study, girl students and the age group of 14-15 reported more headaches and migraines, with statistically significant differences. Many variables can account for the gender disparity: boys and girls, especially in this age range, have differing perceptions of pain. This may be related to Jordanian cultural and social expectations that place more pressure on girls, give them less personal freedom and fewer social choices, in addition to being related to female sex hormones and puberty [28, 37, 38].

In the current study, pulsating pain, unilateralism, and aggravation by physical activity were the most common symptoms of migraine. Ruschel and De Jesus found that migraine was linked to pulsating pain (74%), joint discomfort (58%), and that it intensified with physical activity in 90% of the students who participated [39]. Similar investigations all over the world corroborated our findings Alawneh and, Bataineh, 2006; [16–18, 37].

In the present study, the factors that may increase headache include anxiety and depression (85.7%) and insufficient or inadequate sleep (80.7%), followed by physical exertion and fatigue (75.8%); these results are in line with studies in other countries [23, 40, 41]. The primary migraine triggers differ from person to person. However, stress, particularly the stress associated with school (school activities, bullying, friends), and family difficulties are major triggers of migraine in children and teenagers [1, 2, 10, 42]. Examining what causes stress can help determine which stressors to avoid. A counselor may be required in some circumstances to discover the source of the stress. Regular exercise, appropriate rest and food, and engaging in pleasurable activities and hobbies are all parts of stress management. Insufficient sleep (below the average of eight hours a night) may result in less stamina to deal with stress [1, 2, 10]. Migraine can be caused by changes in usual eating routines or missing meals. Three meals a day, including breakfast, are normal. Weather changes such as storm fronts or changes in barometric pressure can trigger migraine in some people. Migraine can also be triggered by natural hormonal changes induced by the menstrual cycle in teenage girls [1, 2, 10, 27, 34, 37].

### 5.1. Limitations

Rather than the cross-sectional, school-based design used in the current study, a different design might shed more light on this issue, especially on the causal links among the variables, which this study was unable to evaluate. Furthermore, not every adolescent is aware of their family income, history of headaches, and their psychological state. Additional factors such as hormonal changes, dehydration, bullying (school and family), psychological conditions, and substance use may affect the prevalence of headache and migraine.

## 6. Conclusion

According to this study, the prevalence of headache and migraine in Jordan is high and increasing as students grow older. Headache is one of the most common public health issues among high school students aged 12–15, with two-thirds reporting headaches in the previous year, disrupting daily activities and causing them to miss school. It has an impact on their general education as well as their academic achievement and performance. Building and maintaining peer relationships is difficult. Analgesics are being used more frequently, which can lead to misuse. A headache and migraine health education program led by school nurses and other healthcare practitioners is urgently needed.

### 6.1. Implications for School Health

Adolescence is an important step in achieving optimal growth and development and is considered to be one of the most sensitive periods for health and personality building. The world's population now has a greater number and proportion of young people, and providing healthcare to this age group is a critical and ongoing challenge for medical practitioners [43, 44]. Healthcare providers, therefore, need to be prepared to address the adolescent population's health needs. In the early assessment of headache and migraine, information gathering and analysis of the triggering factors are crucial parts of medical treatment. Nurses and healthcare providers play a critical role. They should take part in sophisticated studies to assess the existing state of headache and migraine among adolescents, as well as disseminate healthy solutions that promote children's growth and development, at the same time improving their education and quality of life. Families play a key role in teaching their children to seek medical treatment rather than self-medicate.

Jordan's Ministry of Education and Ministry of Health should collaborate to create a clear strategy and policy for early headache screening, diagnosis, and referral to the necessary medical care.

## Figures and Tables

**Table 1:** Sociodemographic characteristics of adolescents.

Characteristics	No. of students (%)	Mean (SD)
Gender		
Boys	358 (51.7)	
Girls	334 (48.3)	
Total	692 (100%)	
Age (years)		13.9 (1.3)
12	206 (29.8)	
13	190 (27.5)	
14	169 (24.4)	
15	127 (18.4)	
Family monthly income ($)		
<500	320 (46.2)	
500 and more	372 (53.8)	
Mother's education		
Less than secondary education	340 (49.1)	
Secondary and more education	352 (51.9)	
Father's education		
Less than secondary education	199 (28.8)	
Secondary and more education	493 (71.24)	

**Table 2:** Prevalence of headache types among adolescents by gender and age groups.

Sociodemographic characteristics	Headache type					*P* value
	Tension-type headache	Migraine	Unknown	No headache	Total	
Gender						
Girls	40 (11.2)	18 (5.0)	118 (33.0)	182 (50.8)	358 (100)	0.021
Boys	31 (9.3)	15 (4.5)	100 (29.9)	188 (56.3)	334 (100)	
Age groups						
12-13	36 (9.1)	16 (4.0)	103 (26.0)	241 (60.9)	396 (100)	0.032
14-15	35 (11.8)	17 (5.7)	115 (38.9)	129 (43.6)	296 (100)	
Total	71 (10.3)	33 (4.8)	218 (31.5)	370 (53.5)	692 (100)	

**Table 3:** Characteristics of headache among adolescents (*N*^*∗*^ = 322).

Variables	*N* (322)
Time of headache (most of the time)	
Morning	97 (30.1)
Afternoon	225 (69.9)
Pain intensity (most of the time)	
Mild	107 (33.2)
Moderate	160 (49.7)
Severe	55 (17.1)
Associated characteristics (most of the time)	
Throbbing	14 (4.3)
Pressure like	34 (10.6)
Exploding	7 (2.2)
Burning	14 (4.4)
Dullness like	44 (13.7)
Aching	40 (12.4)
Develop gradually	65 (20.2)
Sharp pain	53 (16.5)
Difficult to described	51 (15.8)
Duration of the pain (hours)	
<4	85 (26.4)
4–72	155 (48.1)
>72	82 (25.5)
Affect routine activity	
Yes	105 (32.6)
No	217 (67.4)
Increase school absentees	
Yes	194 (60.2)
No	128 (39.8)
Family history for headache	
Mother	41 (12.7)
Father	43 (13.4)
Brothers or sisters	34 (10.6)
More than one family member	28 (8.7)
No history	167 (51.9)
Do not know	9 (2.8)
Seeking medical help for headache	
Yes	62 (19.3)
No	260 (80.7)
Treatment modality	
Sleeping	216 (67.1)
Eating	17 (5.3)
Drinking	11 (3.4)
Medication	62 (19.3)
Others	16 (5.0)
Advice on using analgesics	
Family	161 (50.0)
School staff	19 (5.9)
Nurse	10 (3.1)
Pharmacists	40 (14.4)
Physician	18 (5.6)
Friends	17 (5.3)
None	33 (10.2)
Others	24 (7.5)
Common symptoms associated with migraine	
Intensity increased with physical activity	34 (68.0)
Pulsating pain	29 (58.0)
Unilateralism	27 (54.0)
Photophobia	21 (42.0)
Phonophobia	11 (22.0)
Vomiting	10 (20.0)
Nausea	7 (14.0)
Others	4 (8.0)

^*∗*^ *N* is the number of adolescents with headache.

**Table 4:** Triggering factors for headaches among adolescents (*N*^*∗*^ = 322).

Triggering factors	Boys (*n* = 176) *N* (%)	Girls (*n* = 146) *N* (%)	Total (*n* = 322) *N* (%)	*P* value
Anxiety and stress	141 (80.1)	135 (92.5)	276 (85.7)	0.032
Physical exertion and fatigue	143 (81.5)	101 (69.2)	244 (75.8)	0.022
Too much or not enough sleep	111 (63.1)	49 (33.6)	260 (80.7)	0.012
Family problems	107 (60.8)	120 (82.2)	227 (70.5)	0.011
Loud noise	115 (65.3)	123 (84.2)	238 (73.9)	0.012
Uncomfortable weather	62 (35.2)	50 (34.2)	112 (34.8)	0.972
Bright lights	41 (23.3)	35 (24.0)	76 (23.6)	0.905
Menstrual cycle	(−)	79 (54.1)	—	—
Others	17 (9.7)	12 (8.2)	29 (9.0)	0.664

^*∗*^ *N* is the number of adolescents with headache.

**Table 5:** Multivariate logistic regression of headache and migraine among adolescents.

Variables	Odd ratio	Confidence interval
Gender		
Girls	1.17	1.07–1.22
Boys	1.00	
Age group		
12-13	1.00	0.99–1.17
14-15	1.15	

## Data Availability

The data used to support the findings of this study are available from the corresponding author upon request.
